# Medical Opinion and Movement

**Published:** 1908-05-30

**Authors:** 


### Medical Opinion and Movement.

The Nauheim cures have been the subject of much criticism on the part of physicians in this country. It is not denied that benefit has frequently followed this treatment in certain cases of cardiac disorder; but the extravagant claims made on its behalf in certain quarters has led to a feeling of scepticism which is characteristic of the mind of the English physician. Nevertheless the Nauheim method of treatment by baths and graduated exercises deserves more attention than is usually given to it. In a paper in *The Medical Record*, Dr. Paul Franze, who is in practice at Bad-Nauheim, gives a short account of the *rationale* of the cures, of the indications for baths and exercises, and of the procedure usually adopted at Nauheim. The author summarises the supposed physiological action of the waters, when applied in the form of baths, without himself endorsing the somewhat fanciful explanation at present in favour. The results of the baths, according to his own observations, are usually a slowing of the pulse, and a rise in blood-pressure in patients whose pressure was previously low, and a fall in the reverse class of cases. This, he asserts, favours the re-establishment of compensation, while the accompanying exercises and massage act in a similar way. The freer flow of blood in the contracting muscles reduces resistance; while the freer reflux of venous blood to the heart, together with the stimulation of the sensory nerve endings, brings about more vigorous contractions of the ventricles and stimulates the heart generally. Dr. Franze speaks with favour of such accessory measures as electric baths and the mineral drinking springs. Certain authorities in England maintain that the Nauheim cure only produces real benefit in cases of functional cardiac disorder. Dr. Franze says that success in Nauheim treatment depends less upon the kind of heart lesion than upon the stage of it: the indications are the early stages of various cardiac lesions. Moreover, the general rules of life laid down by the physicians at Nauheim are an integral part of the treatment, and must be enforced if good is to come. Even when suitable cases are chosen, and the patients faithfully obey orders, there is a proportion of cases in which success is absent. On the other hand, Dr. Frauze states that the benefit of the baths not infrequently manifests itself some time after the conclusion of the treatment.

---

Although "bleeding" is no longer a routine proceeding in the treatment of disease, there are times when such treatment tends to excellent results, and consideration of this fact has led Mendel, in the *Wien. Klin. Wochens.*, to draw up a list of cases in which he believes there are indications for bleeding to be performed. He classifies the cases into two groups: (1) The toxic group, comprising poisoning by carbon monoxide, uræmia, eclampsia, gout, chlorosis, etc.; and (2) the group comprising mechanical obstructions to the circulation, such as valvular lesions of the heart, myocarditis, pneumonia, bronchitis, etc. In the toxic group the effects of bleeding are to diminish the mass of the toxic material, to dilute such toxins as remain by osmosis of fluids from the tissues, and to excite reflexly the vaso-motor system and so regulate the circulation. Bleeding should in most cases be followed by subcutaneous injections of physiological salt solution, supplemented, in the case of carbon monoxide poisoning, by artificial respiration. Mendel claims to have prevented abortion in cases of eclampsia by this means, and also to have relieved acute articular affections, whether of gouty or rheumatic origin. He has great belief in it also as a prophylactic measure in preventing recurrences of the joint affections. Its results upon the victims of chlorosis are, according to him, simply marvellous, especially if stimulating fluids be simultaneously administered by the mouth; the blood-forming organs are stimulated to increased activity thereby.

---

In the second group the result of the treatment is to lower arterial tension by diminishing the volume and viscosity of the blood, and also to relieve venous congestion. Mendel advocates bleeding in apoplexy, whether of the florid type with bounding pulse, red face, and stertorous breathing, or of the pallid type with feeble pulse, pale face, and signs of paralysis of the vaso-motor centres; moreover, it is useful even when signs of paralysis of the limbs are present, since it encourages the resorption of the blood effused. In pneumonia, Mendel advises that the operation be performed as early as possible, so as to relieve pulmonary congestion; he has also found bleeding of use in cases of bronchial asthma, Stokes-Adams disease, capillary bronchitis, and in valvular and myocardial lesions of the heart. As to the quantity of blood that should be abstracted, this should be proportional to the constitution, resistance, and body-weight of the patient. In chlorosis small amounts, from 80 to 90 cubic centimetres of blood should be abstracted at intervals, since the best results are obtained by frequent small abstractions of blood. In poison cases from 200 to 300 cubic centimetres should be taken, the higher figure also denoting the proper quantity to be taken when there is much disturbance of the circulation.

---

Lumbar puncture has been found such a useful measure of relief in a variety of conditions of the central nervous system, that it seemed only reasonable to try its effect in epileptic patients. The convulsive seizures seemed to suggest an increased intracranial tension as a possible cause, the relief of which might be accompanied by amelioration in the condition. Such, however, has not turned out to be the case. Negative results are often as important as positive; it is worth while therefore, to place on record the results of the investigation carried out by Dr. F. Tissot, at the Dury Asylum, on epileptics. He performed lumbar puncture on six epileptics. On each he operated about twice a week, for a period of six to eight weeks, and during the time that these lumbar punctures were made, none of the patients showed any improvement in the condition. The fits recurred at about the same intervals as before and after the treatment, and they often appeared only a few hours after the puncture. The quantity of cerebro-spinal fluid drawn off at a time, usually amounted to about 40 c.c., sometimes as much as 60 or 70 c.c. Dr. Tissot concludes from these experiences, that the hypertension of the cerebro-spinal fluid is the result rather than the cause of the convulsions, and he also suggests that the so-called convulsive substances (especially choline), found in the cerebro-spinal fluid of epileptics, are the products of the affection, and not the determining factors.

EVIDENCE is constantly forthcoming from surgeons in regard to the value of the $x$-rays in the diagnosis of morbid conditions. Sir William Bennett, in an excellent lecture on the subject, emphasises the use of an $x$-ray examination in cases of recurring abdominal pain, simulating appendicitis. He illustrates his lecture by a series of striking cases in which a provisional diagnosis of appendicitis was made, but $x$-ray examination revealed the presence of calculi in the ureters or enlarged calcareous mesenteric glands with adhesions. In cases of actual appendicitis, the $x$-rays may also show the presence of a concretion in the appendix. A further diagnostic power has been found for the $x$-rays, in the determination of a live or still birth. M. Vaillant claims to be able in this way to ascertain separately, whether a dead child has had a separate existence, whether it has breathed, and whether it has taken food. In stillborn children, no organ is visible by radiography. In children who have breathed, the stomach is the first organ visible. In infants who have lived some time, the stomach becomes more visible, and the intestines begin to be defined, and in infants who have taken food, other organs also, such as the lungs, heart, and liver, become visible. In this way M. Vaillant maintains that, within certain limits, it is possible to determine the period of time during which a newly born infant has lived.

HÆMATOPORPHYRINURIA is not a very common condition, but one which it is necessary to be prepared for and ready to detect. It is usually due to intoxication by a drug, notably sulphonal, and also possibly trional or tetronal. When hæmatoporphyrin appears in the urine, after the administration of any of these drugs, it is a sign of danger, and the drugs should be stopped at once. The only sure test for the pigment is the spectroscope. It shows two characteristic absorption bands, one close to, and on the red side of D, and a second half-way between D and E. According to Dr. T. K. Munro, hæmatoporphyrin may occur in the urine independently of intoxication by the drugs above mentioned. He applies the term "paroxysmal hæmatoporphinuria" to the condition. The urine becomes variously tinted pink, red, brownish, or even black. It may be mistaken for hæmaturia, carboluria, or melanuria, unless careful spectroscopic examination of the urine is made. The patients are generally suffering from some more or less chronic ailment, but the immediate cause for the condition of the urine is at present unexplained. The cases which Dr. Munro cites, in which he found this condition of the urine, are various; one was that of a boy, aged seven, suffering from periodic vomiting and acetonuria, another was a lady, aged thirty-six, with multiple neuritis and a pelvic exudate. Now that special attention has been called to the condition, we may soon learn more in regard to its nature and origin.

THE operation of laryngostomy for the relief of cicatricial stenosis of the larynx and the contraction of that organ forms the subject of a communication by Dr. J. Baratoux, of Paris, to the *Laryngoscope*. Cicatricial laryngeal stenosis and contraction of the larynx in patients wearing tubes or cannulas after tracheotomy or intubation are always a source of embarrassment to the surgeon; and the author claims that laryngostomy as first practised by Ruggi is often successful where all other methods fail. The essence of the procedure is prolonge dexposure of the larynx, and elastic dilatation by means of rubber tubes. The operation as performed by Killian consists of four steps: (1) Laryngo-tracheotomy; (2) dilatation with a red rubber drain, lubricated with vaseline, which is changed at first every second day and afterwards at longer intervals; (3) the autoplasty; (4) the maintenance of a provisional opening in the trachea for safety's sake. The details are too elaborate to be dealt with here. The contra-indications to laryngostomy for cicatrical strictures, according to Sargnon and Barlatier, who have already had considerable experience of this operation, are fever, bronchitis, and the presence of pus in the trachea. After the removal of the cannula the voice usually improves to a considerable extent, and the breathing and the general health are restored. The operation necessitates long and delicate after-treatment. For some time the patient needs extremely careful watching, and there are, of course, grave risks of bronchial and pulmonary complications during the first few days. But in competent hands great benefit has resulted from the operation, and cases otherwise incurable have been relieved.

NITROGLYCERINE in the treatment of neuritis is advocated by Burton Stevenson in the *Medical Record*. He reports on thirty-two cases of neuritis treated in this manner. Of these, no fewer than twenty were traced to influenza as a cause. Cases of neuritis due to pressure or injury are not included. Nitroglycerine was administered to the thirty-two cases in the following routine manner: "Beginning with grain 1-100 every eight hours, the interval was reduced one hour in every twenty-four until the full physiological action of the drug was manifest or the patient was taking grain 1-100 every three hours, at which interval it was continued. The disagreeable flushing and headache were controlled by small doses of sodium bromide. When an idiosyncrasy was marked the interval between the doses was lengthened.'' In acute cases (those of less than ten days' standing) benefit resulted within forty-eight hours, and complete relief was obtained in less than a week. In less acute cases the response was slower, but recovery occurred in about a fortnight. In diabetic cases the polyuria and glycosuria were reduced when the drug was used. In chronic cases—that is to say, those of more than three months' standing—the benefit was less striking, but some improvement occurred even in these patients. Here the nitroglycerine was supplemented by progressively increasing doses of ammonium and potassium iodide and the use of the actual cautery over the course of the affected nerve. The author suggests that nitroglycerine, by dilating the arterioles of the sheath and nerve, improves nutrition and hastens absorption of inflammatory deposits.

---

Pulmonary tuberculosis, more especially from the point of view of early diagnosis, is dealt with in a series of six articles by different writers in the current number of the *New York State Journal of Medicine*. In the State of New York the annual death-rate from tuberculosis shows a remarkable uniformity, both as regards the total number of deaths from this cause and their relation to the general death-rate. About 14,000 deaths occur there from tuberculosis in each year, and this number constitutes from one-tenth to one-eleventh of all deaths. Year after year, while the population has increased rapidly, and while the general death-rate has decreased and the prevalence of tuberculosis has also diminished, this level has been retained with little variation. These facts are noted by Dr. John H. Pryor. He considers that the plan of prevention usually employed is haphazard, careless, and ineffectual. "The whole system of prevention, as it exists to-day, is largely a diplomatic game between the physician and the health authorities, and its chief purpose seems to be the encouragement of false security and the study of results instead of causes.'' He rightly regards detection of the disease at the earliest possible moment as the essential feature of any effective scheme for the suppression of tuberculosis. "No consumptive should be allowed to reach a stage when he can communicate the disease to another without an attempt to supply rational relief and segregation.'' Dr. Pryor insists that the victim of early phthisis must be searched for and found before he himself comes for advice, and the place to look for him is among those who have been exposed. "One consumptive should lead us to another. . . . Given an advanced case, an incipient should be traced who is following in the path. The principal places to search are the house and the workshop.'' Repeated, thorough, and prolonged examinations of the chest are necessary. An exposed individual should be instructed in the symptoms which may appear, and the alert physician should insist upon prompt notification of their occurrence. Physical signs must be depended on for early and exact diagnosis. Our full duty is not done if those endangered, especially by prolonged contact, are not kept under close observation.

---

The next article, by Dr. Lawrason Brown, deals more with the methods of early diagnosis in phthisis, and follows logically upon Dr. Pryor's arguments on questions of principle. He believes that a history of prolonged and intimate exposure to infection is of far greater significance than a remote family history of tuberculosis. Phthisis may be a disease of symptoms or of physical signs, and it is dangerous to depend exclusively upon either one or the other. Of the two, symptoms usually arise first. Slight symptoms are here of more importance in early diagnosis than in almost any other serious disease. Lawrason Brown then proceeds to a consideration of the many well-known early symptoms, general and localising, which, when two or more are gathered together, should arouse our suspicions, and call for examination of the sputum when present. He shares the views of the best authorities in this country upon the great danger to the patient of waiting until bacilli appear in the sputum before venturing a positive diagnosis of phthisis. There is much to support Fraenkel's belief that when tubercle bacilli are present in the sputum the disease is no longer in its earliest stage. Brown has little faith in the practical value of examination of the other secretions or of the blood, although he attaches some importance to the examination of the fæces for bacilli in doubtful cases, especially in women, who persistently and against advice swallow their sputum.

---

With regard to physical signs in the chest, the author offers a few practical hints and aphorisms. In most cases of early phthisis râles are heard only during full inspiration, following cough. Râles heard on quiet breathing indicate that the disease is past incipiency. Auscultation is the most reliable method of examination; next in value comes inspection, especially when it discloses a difference in movement of the two sides; but when this contradicts auscultation the latter is the safer guide. Percussion is most important along the upper anterior border of the trapezius while the patient stands relaxed with head straight. The suspicious areas of the lungs should always be examined first. If signs occur at one apex, the patient should be looked upon as tuberculous until the contrary be proved; but signs at one or both of the bases should in like manner be presumed to be non-tuberculous. Myoidema is merely suggestive; but persistent laryngeal pallor, enlarged cervical glands, or scars of old adenitis are of real value. Tuberculin as a means of diagnosis should be used only as a last resort. As for Calmette's test, "in its very simplicity lies its danger; the wholesome dread of the subcutaneous test has always been in its favour and has deterred many from using it rashly or unnecessarily.''

